# Haematological quality and age of donor blood issued for paediatric transfusion to four hospitals in sub‐Saharan Africa

**DOI:** 10.1111/vox.12764

**Published:** 2019-03-05

**Authors:** Sophie Uyoga, Ayub Mpoya, Peter Olupot‐Olupot, Sarah Kiguli, Robert O. Opoka, Charles Engoru, Macpherson Mallewa, Neil Kennedy, Bridon M'baya, Dorothy Kyeyune, Benjamin Wabwire, Imelda Bates, Diana M. Gibb, Ann Sarah Walker, Elizabeth C. George, Thomas N. Williams, Kathryn Maitland

**Affiliations:** ^1^ KEMRI/Wellcome Trust Research Programme Kilifi Kenya; ^2^ Mbale Clinical Research Institute Mbale Uganda; ^3^ Faculty of Health Sciences Busitema University, Mbale Campus Mbale Ugandas; ^4^ Department of Paediatrics Mulago Hospital Makerere University Kampala Uganda; ^5^ Department of Paediatrics Soroti Regional Referral Hospital Soroti Uganda; ^6^ Department of Paediatrics and Child Health College of Medicine University of Malawi Blantyre Malawi; ^7^ School of Medicine Dentistry and Biomedical Science Queen's University Belfast UK; ^8^ Malawi Blood Transfusion Service Blantyre Malawi; ^9^ Uganda Blood Transfusion Service Kampala Uganda; ^10^ Mbale Regional Blood Bank Mbale Uganda; ^11^ Liverpool School of Tropical Medicine and Hygiene Pembroke Place Liverpool UK; ^12^ MRC Clinical Trials Unit Institute of Clinical Trials & Methodology University College London London UK; ^13^ Department of Medicine St Mary's Campus Imperial College London UK

**Keywords:** anaemia, blood transfusion services, donor blood pack, haematocrit, haemoglobin

## Abstract

**Background and Objectives:**

Paediatric blood transfusion for severe anaemia in hospitals in sub‐Saharan Africa remains common. Yet, reports describing the haematological quality of donor blood or storage duration in routine practice are very limited. Both factors are likely to affect transfusion outcomes.

**Materials and Methods:**

We undertook three audits examining the distribution of pack types, haematological quality and storage duration of donor blood used in a paediatric clinical trial of blood at four hospitals in Africa (Uganda and Malawi).

**Results:**

The overall distribution of whole blood, packed cells (plasma‐reduced by centrifugation) and red cell concentrates (RCC) (plasma‐reduced by gravity‐dependent sedimentation) used in a randomised trial was 40·7% (*N* = 1215), 22·4% (*N* = 669) and 36·8% (*N* = 1099), respectively. The first audit found similar median haematocrits of 57·0% (50·0,74·0), 64·0% (52·0,72·5; *P *=* *0·238 vs. whole blood) and 56·0% (48·0,67·0; *P *=* *0·462) in whole blood, RCC and packed cells, respectively, which resulted from unclear pack labelling by blood transfusion services (BTS). Re‐training of the BTS, hospital blood banks and clinical teams led to, in subsequent audits, significant differences in median haematocrit and haemoglobins across the three pack types and values within expected ranges. Median storage duration time was 12 days (IQR: 6, 19) with 18·2% (537/2964) over 21 days in storage. Initially, 9 (2·8%) packs were issued past the recommended duration of storage, dropping to 0·3% (*N* = 7) in the third audit post‐training.

**Conclusion:**

The study highlights the importance of close interactions and education between BTS and clinical services and the importance of haemovigilance to ensure safe transfusion practice.

## Introduction

The availability of safe blood for transfusion is fundamental for every healthcare system. Quality‐assurance practices are a legal requirement for blood transfusion services (BTS) in high‐income countries to minimize patient risk. Yet, even within this context the prolonged storage of donor blood remains controversial 1, 2, 3, since transfusions given to critically ill patients with longer storage age have resulted in unintended, adverse consequences 1, 4, 5. In sub‐Saharan Africa (sSA), where the demand for transfusion is high, little research has been conducted on the quality and safety of donor blood or how these effect post‐transfusion outcomes. With regard to the safety of blood, most studies or reviews have focused on the risks of transfusion‐transmitted infections (TTIs) 6, with a smaller number investigating the microbiological hazards 7. To our knowledge, no previous studies have been conducted within sSA that have investigated the haematological quality or storage age of donor blood under routine operational conditions.

**Figure 1:**
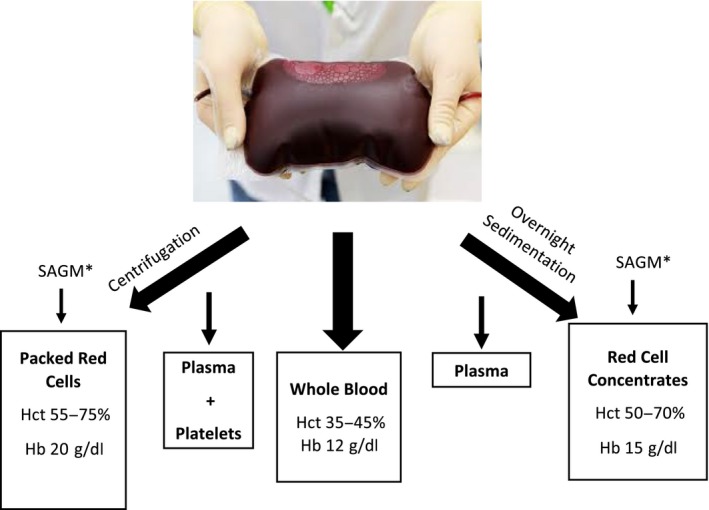
Summary of the blood preparation process and expected haemoglobin and haematocrit values. [Colour figure can be viewed at wileyonlinelibrary.com]

Children are the main recipients of blood transfusions in sSA, where severe anaemia remains a leading cause of both admission to hospital and of direct mortality 8, and is a major factor in the estimated 600 000 malaria deaths each year 9. We are currently investigating the impact of the World Health Organization (WHO) transfusion guidelines on within‐hospital and post‐discharge paediatric survival in a multicentre phase III clinical trial entitled Transfusion and Treatment of Severe Anaemia in African Children: a randomised controlled Trial (TRACT; ISRCTN 84086586) 10. TRACT is being conducted at four hospitals in sSA: three in Uganda (Mulago National Referral Hospital (NRH) and the Mbale and Soroti Regional Referral Hospitals (RRH)) and at one hospital in Malawi (Queen Elizabeth Central Hospital in Blantyre). One intervention that TRACT is investigating is a higher vs. a standard volume of transfused blood (calculated in millilitres per kilogram of the child's bodyweight). In theory, the volume of blood given should translate to a predictable red cell mass being transfused. However, we were unable to find any data on haematological quality control to support this assumption in the settings where the trial was being conducted. Moreover, a further challenge to implementing a protocol to ensure clear separation between the two transfusion volume strategies was that BTS supplied three different pack types: whole blood, red cell concentrates (RCC) and packed cells (Fig. 1). We also investigated the storage age of the blood, that is the time from donation to blood transfusion, as concerns have been raised about the quality of blood stored beyond 23 days (‘old blood’) 11, 12 even when blood is leucocyte‐reduced and storage is quality‐assured. In most countries, in sSA donated blood is not leucocyte‐reduced during its processing and maintaining a cold‐chain is often a challenge 13.

Here, we report the results of audits conducted at different time points during the TRACT trial to investigate the haematological quality and storage duration of donor blood packs provided by BTS at the four trial centres at three time points during the course of the TRACT trial. In addition, in order for practising clinicians to be better informed about the transfusions they regularly prescribe we also include a description of processes of preparation of the different pack types available for transfusion, which were integral to the interpretation of the findings of our audits.

## Materials and methods

In Uganda, the processing and supply of donor packs to Mulago NRH were done by Nakasero National BTS while Mbale and Soroti RRHs received supplies from Mbale Regional BTS. In Malawi, Blantyre Regional BTS processed and supplied blood to Queen Elizabeth Central Hospital.

### Quality assurance

The haemoglobin and haematocrit values of all donor blood packs were verified within the trial using an aliquot, collected from the blood line prior to the start of each transfusion. In order to accurately calculate volumes of blood received by children in the trial, all donor packs were weighed before transfusion. The packs were then gently agitated until well‐mixed before being run into a gauged (and filtered) ‘burette giving‐set’ and then through the infusion line to the intravenous infusion or giving‐set. The first few drops of blood released from the giving‐set were collected under sterile conditions into a 2 ml apex tubes before infusion lines were connected to patients’ cannulae. Haemoglobin values were measured at the bedside using the HemoCue Hb301 system (HemoCue AB, Angelholm, Sweden), quality‐controlled with Eurotrol Hb301 Control reagents (Eurotrol, Ede, the Netherlands) to ensure accuracy. Haematocrits were determined by centrifugation in capillary tubes (Hematospin 1300, Hawksley and Sons Ltd). In addition, the clinical team recorded the pack identifier numbers, the pack type as issued by the transfusion laboratories (whole blood, packed cells or RCC), the blood volume, calculated as the weight of the pack minus the weight of the pack type containing no donor blood, and the date of collection from the donor (in order to calculate storage age). Prior to the start of the trial, we conducted training sessions on procedures for collecting this information. We conducted three audits examining donor blood data during the trial: (1) at the beginning of the trial during September 2014–January 2015 (Audit 1); (2) following a period of consultation with the BTS (February–May 2015; Audit 2); and (3) following a period of re‐training during June 2015–January 2016 (Audit 3). The duration of storage of donor blood before transfusion was categorized as short (≤14 days), long (15‐42 days) or expired (>35 days for whole blood and >42 days for RCC and packed cells) 12.

### Statistical analysis

Comparison of haemoglobin and haematocrit levels for the three pack types across the three audits was done using Kruskal–Wallis equality‐of‐populations rank test and Wilcoxon rank‐sum test. All analyses were conducted using Stata v15 (Stata Corp, Timberlake, Memphis, TN, USA).

## Results

A total of 2983 transfusion blood packs were utilized in the trial between September 2014 and January 2016. Through our audits, we found that three pack types produced by local BTS were supplied for use in the trial: (1) whole blood, collected from donors and stored without any preparation; (2) packed cells, produced by centrifugation, to removal platelets and plasma, followed by the addition of sodium, adenine, glucose and mannitol (SAGM) solution; and (3) ‘red cell concentrates’ (RCCs), which were supplied in Uganda only. RCCs were produced by gravity‐dependent sedimentation as an alternative to centrifugation because of limited capacity for mechanical separation. The process of gravity‐dependent sedimentation involved packs being hung at room temperature overnight before plasma was decanted off and SAGM added in a closed system of bags. On the basis of the preparation methods used, packed cells should evidently be the most concentrated followed by RCCs and then whole blood (Fig. 1). The overall distribution of whole blood, packed cells and RCCs included in our three audits was 40·7% (*N* = 1215), 22·4% (*N* = 669) and 36·8% (*N* = 1099), respectively. The first, second and third audit included 330, 606 and 2047 packs, respectively. Haemoglobin and haematocrit values were available for 2970 and 2737 donor packs, respectively.

### Haematological quality of donor blood

#### First audit

**Figure 2:**
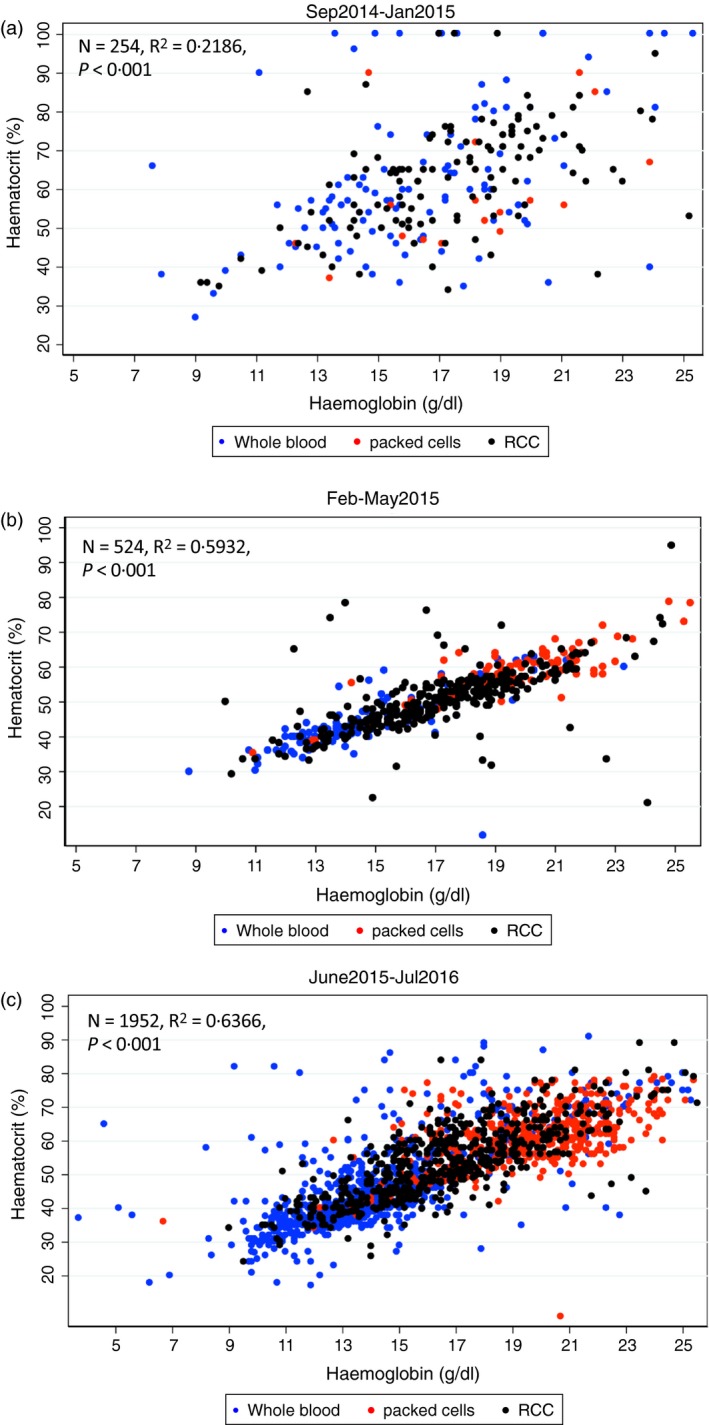
Scatter plots of haemoglobin and haematocrit values for the blood packs used at start of the trial (a), during the training period (b) and post‐training (c). [Colour figure can be viewed at wileyonlinelibrary.com]

In the first audit which was undertaken in Uganda only, we found statistically significant (not explained by chance alone) but only moderate differences of haematological values between the pack types. The median haemoglobin level was 16·4 g/dl (IQR: 14·0, 18·8; *N* = 130) in whole blood packs, 17·3 g/dl (15·4, 19·4; *N* = 136, *P *<* *0·001 vs. whole blood) in RCC and 18·9 g/dl (17·3, 20·6; N = 62; *P *=* *0·025) in packed‐cell packs. Moreover, we found no significant differences between pack types in terms of haematocrit values, the medians being 57·0% (50·0, 74·0), 64·0% (52·0, 72·5; *P *=* *0·238) and 56·0% (48·0, 67·0; *P *=* *0·462) in whole blood, RCC and packed cells, respectively (Table 1 and Fig. 2a). Median haemoglobin and haematocrits values for the whole blood packs were also substantially higher than the expected values of 12 g/dl and 35–45% respectively. Conversely, the medians were lower than those expected (20 g/dl and 55–57% respectively) among packed cell donations (Fig. 1)^14^.

**Table 1:** Summary of median (IQR) haemoglobin and haematocrit in the three audits

	Pack type	Expected values	*N*	Audit 1	*P* a	*N*	Audit 2	*P* a	*N*	Audit 3	*P* a
Hb (g/dL)	WB	12	130	16·4 (14·0, 18·8)	<0·001	132	14·2 (13·0, 16·1)	<0·001	948	13·7 (12·5, 15·3)	<0·001
	RCC	15	136	17·3 (15·4, 19·4)		361	17·2 (15·5 18·9)		600	16·5 (14·7, 18·4)	
	PC	20	62	18·9 (17·3, 20·6)		110	19·5 (18·0, 21·2)		491	19·7 (17·8, 21·2)	
HCT (%)	WB	35–45	111	57·0 (50·0, 74·0)	0·271	127	42·0 (40·0, 47·0)	<0·001	912	41·0 (37·0, 49·0)	<0·001
	RCC	50–70	124	64·0 (53·0, 72·5)		309	50·9 (45·0, 55·3)		564	53·0 (45·0, 61·0)	
	PC	55–75	19	56·0 (48·0, 67·0)		91	58·0 (53·0, 62·0)		481	61·0 (56·0, 66·0)	

B, whole blood; PC, packed cell; RCC, red cell concentrates.

a *P* Kruskal–Wallis equality‐of‐populations rank test.

#### Quality assurance of research methods

Because these results were unexpected, we were concerned that there may have been a methodological flaw in our sampling technique. To better understand the lack the expected differences between the three pack types in terms of their haematological indices, we re‐checked the methods of sample collection, the reliability of our haemoglobin and haematocrit readings and the way in which the pack types were labelled by the BTS. In a sample of 10 donor packs from each of the three Ugandan study centres, we audited the measurement of haemoglobin and haematocrit values against values from automated blood analysers. For sample collection, we compared these measurements in samples of donor blood collected in the prescribed manner (at the point of exit from the giving set) to samples collected from the top of the burette. Neither assay method nor sampling point suggested any methodological explanations for the observed lack of clear haematological differences between donor pack types (Table S1). Consultation with the BTS centres suggested a low emphasis on providing clear labelling instructions for clinicians regarding the pack types. This lead to a subsequent change in pack labelling and our realization of the need for a series of training sessions for BTS and blood bank staff and clinicians (including trial staff) on the importance of pack type identification and labelling.

#### Second and third audits

**Figure 3:**
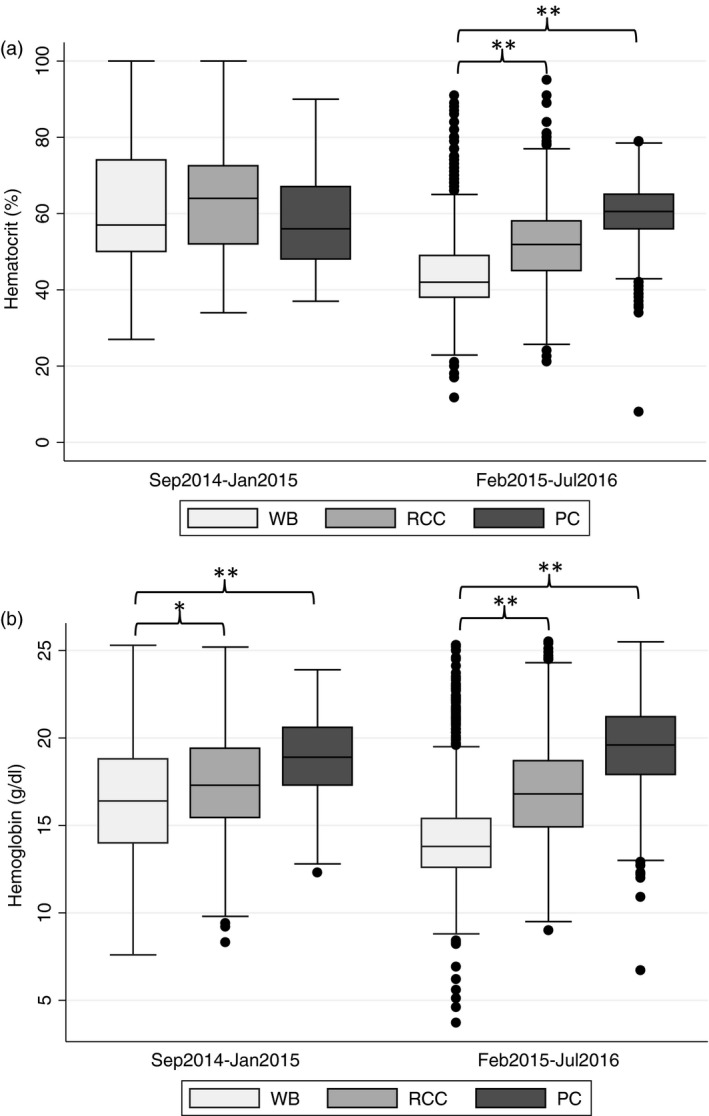
Box plots of haematocrit (a) and haemoglobin (b) before (Sep 2014–Jan 2015) and after training (Feb 2015–Jul 2016) by pack type. PC, packed cells; RCC, red cell concentrates and WB, whole blood). *P < 0·05, ** P < 0·001.

Audit 2, conducted after a period of intense training, included 606 donor packs while Audit 3 involved 2047 donor packs. In both these subsequent audits, we found clear and significant differences between the three pack types in terms of haemoglobin and haematocrit (Table 1 and Fig. 3a,b) and between countries (Table 2). Linear regression analysis showed a clear relationship between pack type and both haemoglobin and haematocrit values in which, as originally expected, the lowest values were seen in whole blood and the highest in packed cells (Fig. 2b,c). Only 5·5% (*N* = 164) of the packs had haemoglobin concentrations that were out of range (two standard deviations above or below the mean for each of the pack types).

**Table 2:** Summary of median (IQR) haemoglobin (g/dl) and haematocrit (%) in audits 2 and 6 stratified by country

Pack type		*N*	Audit 2 Haemoglobin	*P* a	N	Audit 3 Haemoglobin	*P* a
Whole blood	Malawi	71	13·7 (12·8, 14·5)	<0·001	395	13·5 (12·6, 14·3)	<0·001
	Uganda	62	15·2 (13·6, 17·4)		553	14·1 (12·4, 16·5)	
Packed cells	Malawi	10	19·2 (17·2, 19·9)	0·333	83	20·6 (18·9, 21·5)	0·003
	Uganda	100	19·5 (18·0, 21·2)		408	19·4 (17·7, 21·1)	

Pack type		*N*	Audit 2 Haematocrit	*P* a	*N*	Audit 3 Haematocrit	*P* a
Whole blood	Malawi	73	41·0 (38·0, 47·0)	<0·001	386	39·0 (36·0, 41·0)	<0·001
	Uganda	54	47·1 (42·0, 55·0)		526	46·0 (40·0, 57·0)	
Packed cells	Malawi	9	55·0 (49·0, 57·0)	0·101	79	56·0 (53·0, 59·0)	<0·001
	Uganda	82	59·0 (54·1, 62·0)		402	62·0 (57·5, 68·0)	

a *P* – Wilcoxon rank‐sum test for comparison by pack type between the two countries.

### Storage time

Data on the age (or storage time) for 2964 donor packs were collected prospectively throughout the TRACT trial. Storage time was highly heterogeneous, with the median age of transfused blood being 12 days (IQR: 6, 19) and a range of 0–77 days. Overall, 18·2% (*N* = 537) of the blood used in the trial was classified as old (above 21 days of age, Table 3). The median age of blood before training (Audit 1) was 15 (IQR: 10–21) days and after training (Audits 2 & 3) was 11 (IQR: 6–18) days (*P *<* *0·001). Through our audits, we demonstrated that 9 (2·8%) blood packs used earlier in the trial (Audit 1) were issued past the recommended duration of storage. This proportion reduced to 1·0% (*N* = 6) and 0·3% (*N* = 7) in the Audits 2 and 3, respectively.

**Table 3:** Storage duration profiles of blood packs used in the trial

Duration of storage	Audit 1	Audit 2	Audit 3	Total
Short (0–14 days)	99 (30·5)	162 (26·9)	940 (46·1)	960 (40·5)
Long (15–42 days)	216 (66·7)	435 (72·1)	1092 (53·6)	1733 (58·8)
Expired^a^	9 (2·8)	6 (1·0)	7 (0·3)	22 (0·7)
Total	324	603	2039	2956

*N* (%).

a Expired (>35 days for whole blood and >42 days for red cell concentrates and packed cells).

## Discussion

One of the key findings of this study was that, unexpectedly, despite US President's Emergency Plan for AIDS Relief (PEPFAR) strengthening of the Ugandan BTS which included the introduction of prepared RCC, Audit 1 demonstrated negligible differences in haematological indices between the three pack types. Suboptimal pack labelling in Uganda resulted in the early part of the trial having some inadvertent non‐adherence to ‘blood volume’ (a proxy for red call mass) randomisation strategies. Reviewing all prescribed packs, and identifying the correct pack type as opposed to what the clinician had inadvertently believed had been provided, during the first 4 months of the trial, non‐adherence to randomised transfusion volume was approximately 77% following the protocol, although at the time of the transfusion clinicians’ prescriptions were correct for the blood volume based on what they understood had been provided. Since then we observed improvement in protocol adherence following training with non‐adherence to randomised blood volumes between February and May 2015 reduced to 7%.

A second finding during the initial audit period was that 2·8% of blood prescribed had exceeded its recommended storage period. Consultation between the BTS and clinical teams lead to changes to standard operating procedures used by the BTSs and hospital transfusion laboratories was subsequently implemented. This resulted in improved quality of blood. As evidenced by subsequent audits, we were able to demonstrate significant difference in haemoglobin and haematocrit of the three pack types after the consultation and training and the proportion of ‘expired blood’ used by the clinical teams was also significantly reduced.

For more than a decade, driven by safety concerns that blood transfusions may have been a factor in HIV transmission, multilateral donors including PEPFAR have provided technical and financial support to national BTS in low‐income countries in sSA, with a view to improving the quality‐assured screening of all donated blood for transfusion‐transmissible infections. This has greatly increased blood supply and reduced transfusion‐transmissible infections, as previously reported 15, 16. However, the implementation of such recommendations has also resulted in the adoption in these countries of BTS practices and policies from high‐income countries, including the centralization of BTSs and the preparation of components in place of the use of whole blood. Concerns have been raised that centralization may adversely impact on the equitable and timely access to blood transfusion 17. Moreover, component preparation policies are costly and time‐consuming and result in delays between collection from donors and supply to local blood banks, thus increasing the storage times of blood. Finally, although there appears to be a mismatch between transfusion guidelines incorporating recommendations for standard volumes of whole blood and packed cells (median donor haemoglobin ~20 g/dl), the same does not seem to be true but for RCCs (median donor haemoglobin ~15 g/dl) which in some countries are the largest proportion of donor blood pack now being issued from PEPFAR‐compliant BTSs. This inconsistency between guidelines and donor packs in use has implications for the volume and number of packs of blood required to achieve a similar degree of haemoglobin correction in a recipient and might therefore impact on clinical outcomes, including survival. In the case of paediatric transfusion, WHO recommends a standard volume for transfusion (20 mls/kg of whole blood or 10 mls/kg of packed cells) for all children with profound (Hb < 4/g/dl) or severe and complicated (Hb < 6/g/dl plus one or more severity features) anaemia, but gives no guidelines for RCCs. Thus, the increasing production of RCCs by BTSs may result in many children being initially under‐transfused, increasing the need for re‐transfusion, an issue observed in a previous study 18. Worldwide there is little evidence to support the exclusive use of red cell concentrates, which in our experience were a third of blood packs issued by BTS. Most blood products used in hospitals in Africa are generally for emergency transfusion 19, to replacing lost volume (pregnancy‐related emergencies) and trauma or paediatric transfusion for life‐threatening anaemia, for which paediatric guidelines suggest are better treated with whole blood than blood components 17, 20.

Numerous challenges face BTS in sSA, including lack of adequate finance; adequate infrastructure; safe blood donors; unbroken supply of consumables and reagents, limited man‐power to process collected blood, stock outs of screening reagents, lack of transport for transportation of packs to regional blood banks and regular power‐outages. Such challenges threaten the safe preparation of RCCs by gravity‐dependent sedimentation, and also result in suboptimal storage conditions. Moreover, processing at regional BTS leads to delays between donation, processing and distribution across the regional hospital services which can contribute to prolonged storage times. Prior to the trial beginning, we believed that pressure from demand would mean that ‘old blood’ would be rare. Instead, we found that the age of blood supplied during TRACT was quite diverse, and included some packs that should have been discarded as they were ‘expired**’**. Prolonged storage may result in adverse consequences owing to red cell changes (the storage lesion) that may render them less effective as oxygen carriers and lead to an accumulation of metabolites and cytokines that may lead to untoward side effects 21, 22. The TOTAL trial, conducted in 270 Uganda children investigated whether leucocyte‐reduced RCCs stored for 25–35 days lead to worse outcomes than packs stored for 10 or less days. Lactate clearance, the primary endpoint, was not inferior in those receiving prolonged compare to short storage donor blood, as were all other secondary endpoints including mortality 23. Educating both BTS staff and clinicians to not issue or use blood transfusions that have gone beyond their expiry date is clearly important. In this respect, it is also worth noting that in the African health system, there is little formal training of clinicians in haemovigilance and there are no reporting systems similar to the SHOT (serious hazards of transfusion) scheme in the UK 24. Thus, despite being a major issue for research in other parts of the world, there remains uncertainty over whether prolonged storage of non‐leucocyte reduced blood under standard storage conditions impacts on patient outcome in SSA.

Finally, it is important to point out that donated blood issued for transfusion by BTSs or hospitals blood banks in sSA is likely to have several differences compared with high‐income countries. Beyond the issues of quality assurance and lack of leucocyte depletion are the genetic diversity of donors, as many have inherited red cell disorders (including α‐thalassaemia, sickle cell trait and glucose‐6‐phosphate dehydrogenase deficiency) 25, 26, all of which are associated with shorter red cell half‐lives. As a result, this requires locally generated research studies to help African health services refine their blood donation practices and policies. We suggest that the findings of our first audit reflect the reality of paediatric transfusion across similar settings that have transitioned from the exclusive use of whole blood to include a majority of packs prepared as RCCs, which are not currently recognized in treatment guidelines, nor has there been a concerted effort to inform clinicians about how this may affect transfusion practice.

## Funding

Medical Research Council, and DFiD (thorough a concordat) United Kingdom Trial Grant MR/J012483/1

## Conflict of interest

The authors declare that they have no conflicts of interest relevant to the manuscript submitted to Vox Sanguinis.

## Author contributions

POO, SK, ROO, MM, NK were responsible for acquisition of the data. BM, DK and BW were responsible for coordination of blood transfusion service, training and review and interpretation of the donor pack data. S.U., E.C.G., A.M., S.W., T.N.W. IB and K.M. reviewed and managed the project and analysed the data provided. All authors contributed to the design of the study and review of the manuscript. S.U., T.N.W. and K.M. wrote the initial draft of the paper.

## Supporting information


**Table S1** Comparison of sample collection and analysis methods at three TRACT trial sites in Uganda.
