# Supplementary material for: Haematological quality and age of donor blood issued for paediatric transfusion to four hospitals in sub‐Saharan Africa
Source: Vox Sang. 2019 Mar 5;114(4):340–8. doi: 10.1111/vox.12764 (PMC6563499; doi:10.1111/vox.12764)
Supplement: Supplementary file 1 — Table S1 Comparison of sample collection and analysis methods at three TRACT trial sites in Uganda. [file VOX-114-340-s001.docx]

**SUPPLEMENTARY TABLES**

**Table SI.** Comparison of sample collection and analysis methods at three TRACT trial sites in Uganda.

|  |  | **Haemocrit:**  **Centrifuge** | | ***P**** | **Haemocrit:**  **CBC** | | | ***P**** | | **Haemoglobin**  **CBC** | ***P**** |
| --- | --- | --- | --- | --- | --- | --- | --- | --- | --- | --- | --- |
| Mbale (N=10) | Upper (burette) | 49.7 (2.1) | | 0.64 | 45.3 (2.5) | | | 0.87 | | 13.4 (0.8) | 0.28 |
|  | Lower (Line) | 49.4 (1.9) | |  | 45.4 (1.5) | | |  | | 13.4 (0.8) |  |
| Mulago (N=10) | Upper (burette) | 49.5 (2.17) | | 0.86 | 59.8 (1.8) | | | 0.41 | | 18.4 (0.5) | 0.13 |
|  | Lower (Line) | 49.1 (1.88)** | |  | 59.9 (1.9) | | |  | | 18.4 (0.5) |  |
| Soroti (N=10) | Upper (burette) | 51.7 (0.9) | | 0.72 | 49.0 (0.9) | | | 0.36 | | 14.3 (0.2) | 0.52 |
|  | Lower (Line) | 51.3 (1.3) | |  | 48.3 (1.4) | | |  | | 14.2 (0.2) |  |
|  |  |  |  | | |  |  | |  | |  |

**Mean (standard Error); *P** Student’s *t* - test; CBC - complete blood count.**

**** The lower than expected hematocrit result by the centrifuge than that by the CBC machine, which resulted in the decommissioning and replacement of the centrifuge.**
